# Case Report: Selective metal cell excision during open TAVR implantation preserves coronary access while maintaining valve integrity and function

**DOI:** 10.3389/fmed.2026.1840009

**Published:** 2026-05-29

**Authors:** Julia E. Katter, John Paul Tannous, Katherine A. Roberts, Shawn M. Ahmad, Michael N. Young, Henry J. Tannous

**Affiliations:** 1 Geisel School of Medicine at Dartmouth, Hanover, NH, United States; 2 Quinnipiac University, Hamden, CT, United States; 3 Dartmouth-Hitchcock Medical Center, Lebanon, NH, United States; 4 Mt. Ascutney Hospital and Health Center, Windsor, VT, United States

**Keywords:** cardiac surgery, case report, coronary access, TAVR, valve degeneration

## Abstract

**Background:**

Coronary access following transcatheter aortic valve replacement (TAVR) remains challenging and should be carefully considered when planning re-intervention for patients with structural valve degeneration. Current management options [valve-in-valve (ViV) TAVR or surgical aortic valve replacement and TAVR explant] both carry significant benefits and limitations.

**Case presentation:**

A 78-year-old female with a degenerated 23 mm Sapien 3 TAVR presented with severe prosthetic stenosis, moderate paravalvular leak (PVL), and progressive dyspnea. A standard TAVR ViV had a suboptimal risk of not addressing the PVL and worsening prosthesis-patient mismatch, whereas TAVR explantation and SAVR with root enlargement carried an increased surgical risk.

**Intervention:**

Open surgical TAVR explantation with direct re-implantation of a 26 mm Sapien Ultra valve, combined with selective metal cells excision facing the left main coronary artery (LMCA) and felt patch repair of the PVL.

**Outcome:**

The patient achieved immediate resolution of severe stenosis and elimination of PVL, with sustained clinical improvement at six-month follow-up and a more favorable coronary access.

**Conclusion:**

Selective metal cell excision during open TAVR implantation is a technically feasible approach to prophylactically preserve coronary access while maintaining valve integrity and function. This technique may benefit high-risk patients requiring TAVR explants and root enlargement SAVRs.

## 1 Introduction

Coronary access after transcatheter aortic valve replacement (TAVR) represents a significant clinical challenge as TAVR becomes increasingly prevalent and extends to younger patients who may require future coronary interventions. Aortic stenosis commonly occurs concurrently with coronary artery disease (CAD), with rates as high as 65% in patients undergoing TAVR (1, 2). While documented rates of percutaneous coronary intervention (PCI) following TAVR range from 0.9 to 2%, risk increases substantially in patients with known CAD and multivessel disease (3, 4).

What is Unique About This Case: We present a case of selective removal of TAVR metal cells facing the left main coronary artery (LMCA) during valve implantation to address the critical concern of impaired coronary access post-TAVR. Frame modification was performed post-deployment to preserve coronary access for future interventions while maintaining hemodynamic stability and the procedural advantages of TAVR.

Current re-intervention strategies for degenerated TAVR include valve-in-valve (ViV) TAVR or TAVR explantation followed by surgical aortic valve replacement (SAVR). However, ViV TAVR can worsen prosthesis-patient mismatch (PPM) due to the cumulative effect of multiple prosthetic layers plus the native valve. This strategy would not definitively address the existing paravalvular leak and can impede future coronary access (5–8). The other strategy, SAVR following TAVR explantation, carries a 13–20% mortality rate, and an increased operative complexity when combined with root enlargement (9–11).

Given the limitations of existing methods, our case presents a novel approach, selective metal cell excision during open TAVR implantation, that is feasible and does not compromise valve function.

## 2 Case description

A 78-year-old female with a significant cardiac history presented to our institution with progressive dyspnea and acute decompensated heart failure. Two years prior, she had undergone successful transcatheter aortic valve replacement with a 23 mm Sapien 3 valve for severe aortic stenosis, which had initially provided symptomatic relief and improved functional capacity.

Her past medical history was notable for several comorbidities that complicated her presentation. In addition to the prior TAVR for aortic stenosis, she had non-obstructive coronary artery disease, chronic kidney disease, obesity (BMI 46.2 kg/m^2^; BSA 2.36 m^2^), and recent deconditioning related to repeated heart failure exacerbations. Her index TAVR was performed in April 2022 with a 23 mm Sapien 3 Ultra, guided by CT demonstrating an aortic annulus of 21.6 × 27.8 mm (area 479 mm^2^; circumference 81 mm), sinotubular junction of 29.6 × 29.6 mm, sinus of Valsalva of 33.2 mm, and annulus-to-coronary heights of 13.8 mm on the left and 11.8 mm on the right, with a severe focal calcific arc (~90°) extending from beneath the commissure between the left and noncoronary leaflets into the LVOT. At the time of her index procedure, her STS Predicted Risk of Mortality was 8.4%, placing her in the high-risk category and supporting the initial decision to pursue TAVR over SAVR. The procedure was initially successful, with postoperative transthoracic echocardiography demonstrating an effective orifice area (EOA) of 2.04 cm^2^ (indexed EOA 0.86 cm^2^/m^2^), peak gradient of 17 mmHg, and mean gradient of 10 mmHg, with no paravalvular regurgitation, confirming a well-functioning prosthesis without patient–prosthesis mismatch at implantation. Overall, these comorbidities collectively placed her in a higher-risk category for complex cardiac surgery.

Upon admission, the patient presented with progressive dyspnea that had worsened over several weeks. She was found to be in acute decompensated heart failure with associated hypoxic respiratory failure and acute-on-chronic renal insufficiency, necessitating hospitalization and intensive management. Physical examination revealed findings consistent with volume overload, including peripheral edema, elevated jugular venous pressure, and hepatomegaly. Her central venous pressure was elevated at approximately 20–22 mmHg, indicating severe right heart strain and venous congestion.

Auscultation revealed significant pulmonary crackles bilaterally, reflecting pulmonary edema. The patient’s functional status had deteriorated significantly, limiting her to minimal activity due to severe dyspnea.

Diagnostic investigations were performed to characterize the etiology of her acute decompensation. Cardiac catheterization confirmed that the acute presentation was not due to acute coronary syndrome. The left main was disease-free; the LAD showed mild diffuse disease (≤25%) with a 35% mid-segment stenosis and a 75% discrete stenosis in the ostial segment of a moderately-sized second diagonal branch; the LCX and RCA each showed mild diffuse disease (≤25%). No lesions met criteria for revascularization. The absence of obstructive left main or proximal LAD disease reinforced the importance of preserving future coronary access, directly informing our decision to selectively excise the metal cells facing the left main coronary artery at re-implantation. Right heart catheterization revealed severely elevated pulmonary arterial pressures (72/35 mmHg preoperatively; 65/30 mmHg intraoperatively) with a preserved cardiac index of 2.78 L/min/m^2^ and a pulmonary vascular resistance of 130 dyn·s·cm^−5^ (1.6 Wood units) by estimated Fick technique. The normal PVR indicated predominantly post-capillary pulmonary hypertension reflecting left heart disease rather than fixed pulmonary vascular remodeling.

**Figure 1:**
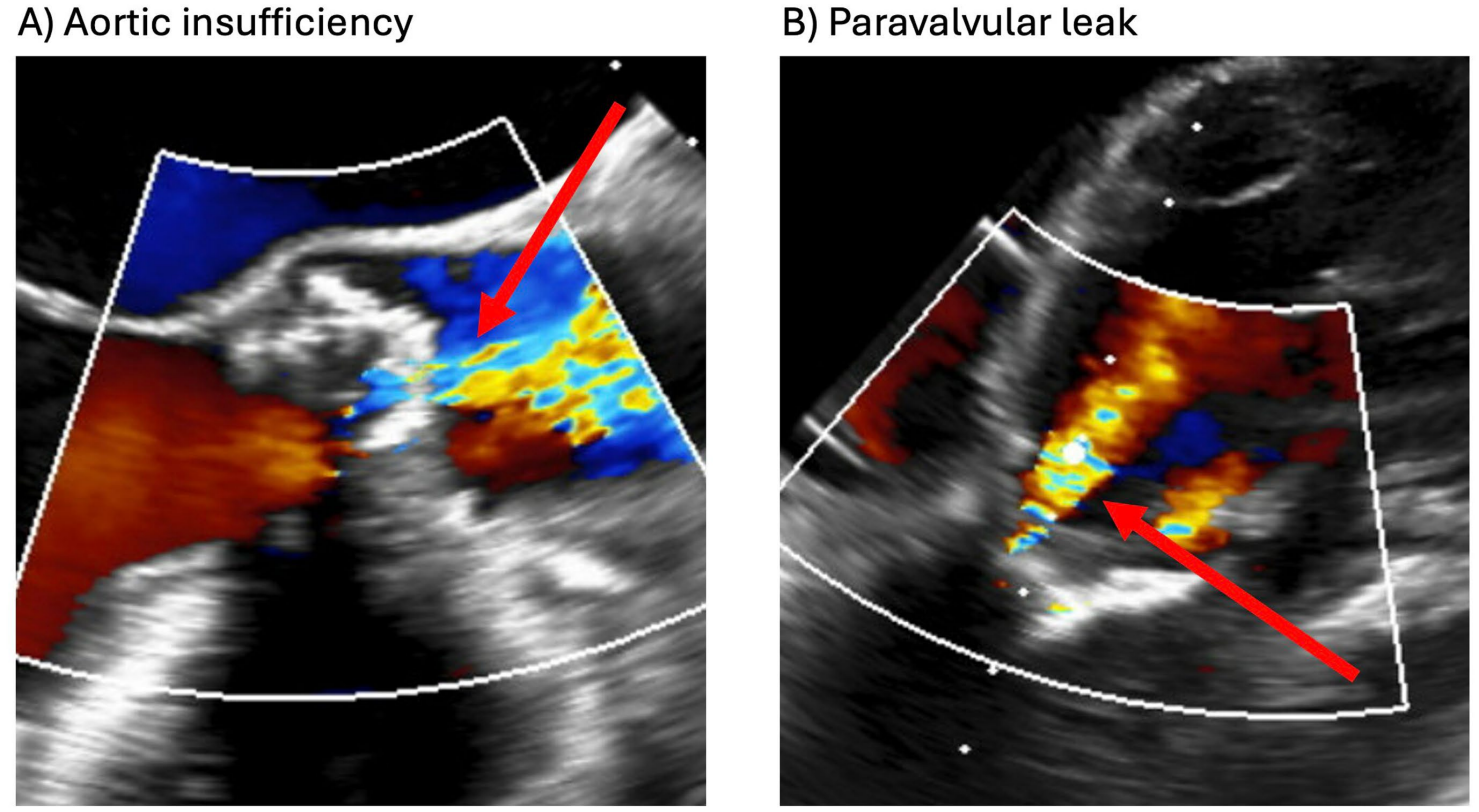
Preoperative TEE demonstrating **(A)** prosthetic valve stenosis and **(B)** paravalvular leak.

Preoperative transthoracic echocardiography demonstrated a preserved left ventricular ejection fraction of 65%, indicating that her decompensation was due to valvular dysfunction rather than primary myocardial failure. The prosthetic aortic valve showed severe stenosis (EOA 0.5 cm^2^; mean gradient 66 mmHg) with a moderate paravalvular leak (PVL), alongside moderate mitral and tricuspid regurgitation (Figure 1), a marked decline from the post-implantation baseline (EOA 2.04 cm^2^; iEOA 0.86 cm^2^/m^2^; mean gradient 10 mmHg) over approximately 2 years, consistent with early structural valve degeneration rather than progressive patient–prosthesis mismatch. Intraoperative transesophageal echocardiography corroborated these findings (EOA 0.6 cm^2^; iEOA 0.25 cm^2^/m^2^; mean gradient 46 mmHg; moderate PVL).

Cardiac CT guided operative planning. The indwelling stent showed internal diameters of 19 × 19 mm with mild leaflet calcification and no thrombus. The native annulus measured 18.6 × 22.7 mm (area 330 mm^2^), with a sinotubular junction of 28.5 × 29.8 mm, sinus of Valsalva width of 33.2 mm, and annulus-to-coronary heights of 11.1 mm (left) and 10.6 mm (right). The constrained 19 mm stent diameter predicted meaningful patient–prosthesis mismatch with a valve-in-valve strategy, while favorable coronary heights and sinus dimensions supported explantation and upsizing to a 26 mm Sapien Ultra under direct visualization.

## 3 Care timeline

Clinical timeline of case presentation and intervention.

**Table 1:** 

Timepoint	Key finding
2 years prior	23 mm Sapien 3 implanted
Weeks prior	Worsening dyspnea
Admission	
Acute presentation	CHF, hypoxic respiratory failure, AKI on CKD
Physical exam	CVP 20–22 mmHg; volume overload
Cardiac catheterization	Non-obstructive CAD; PA 72/35 mmHg
Preop echo	EF 65%; severe AS (EOA 0.5 cm^2^, MG 66 mmHg); Moderate PVL, MR, TR
Operative	
Intraoperative echo	AS confirmed (65/30 mmHg)
TAVR explantation	23 mm Sapien 3 removed; thickened, stenotic leaflets
PVL repair	5 mm × 25 mm felt patch placed at aortomitral curtain
New valve deployment	26 mm Sapien Ultra implanted; coronary alignment verified
Novel technique	Metal cells facing LMCA selectively excised; coronary access confirmed
Tricuspid repair	MC3 annuloplasty band placed
Cross-clamp time	88 min
POD 0: Extubation	Successful; stable respiratory status
POD 7: Arrythmia	Paroxysmal AFib with bradycardia; permanent pacemaker placed
POD 8: Discharge	Home in stable condition
6 months follow-up	
Follow-up echo	Well-functioning valve; trace TR; improved MR; no stenosis
Clinical status	Resolution of dyspnea; improved functional capacity

AKI, Acute kidney injury; CKD, Chronic kidney disease; CVP, Central venous pressure; EF, Ejection fraction; MR, Mitral regurgitation; PA, Pulmonary artery; PVL, Para-valvular leak; TR, Tricuspid regurgitation

## 4 Diagnostic assessment

Standard re-intervention options were considered and deemed suboptimal or too risky. Valve-in-valve TAVR would worsen the patient’s existing PPM, would not definitively address the calcified PVL, and could impede future coronary access. SAVR with root enlargement would increase intraoperative risk secondary to her age and comorbidities. The patient’s elevated baseline surgical risk (STS PROM 8.4% at index TAVR) and her clinical decline, with worsening heart failure, acute-on-chronic kidney injury, pulmonary hypertension, and severe obesity, further supported a strategy that minimized cardiac ischemia time and avoided the additional complexity of root enlargement. The multidisciplinary team selected open surgical TAVR explantation with direct re-implantation of a larger transcatheter valve combined with targeted PVL repair and selective metal cell excision to preserve coronary access.

**Figure 2:**
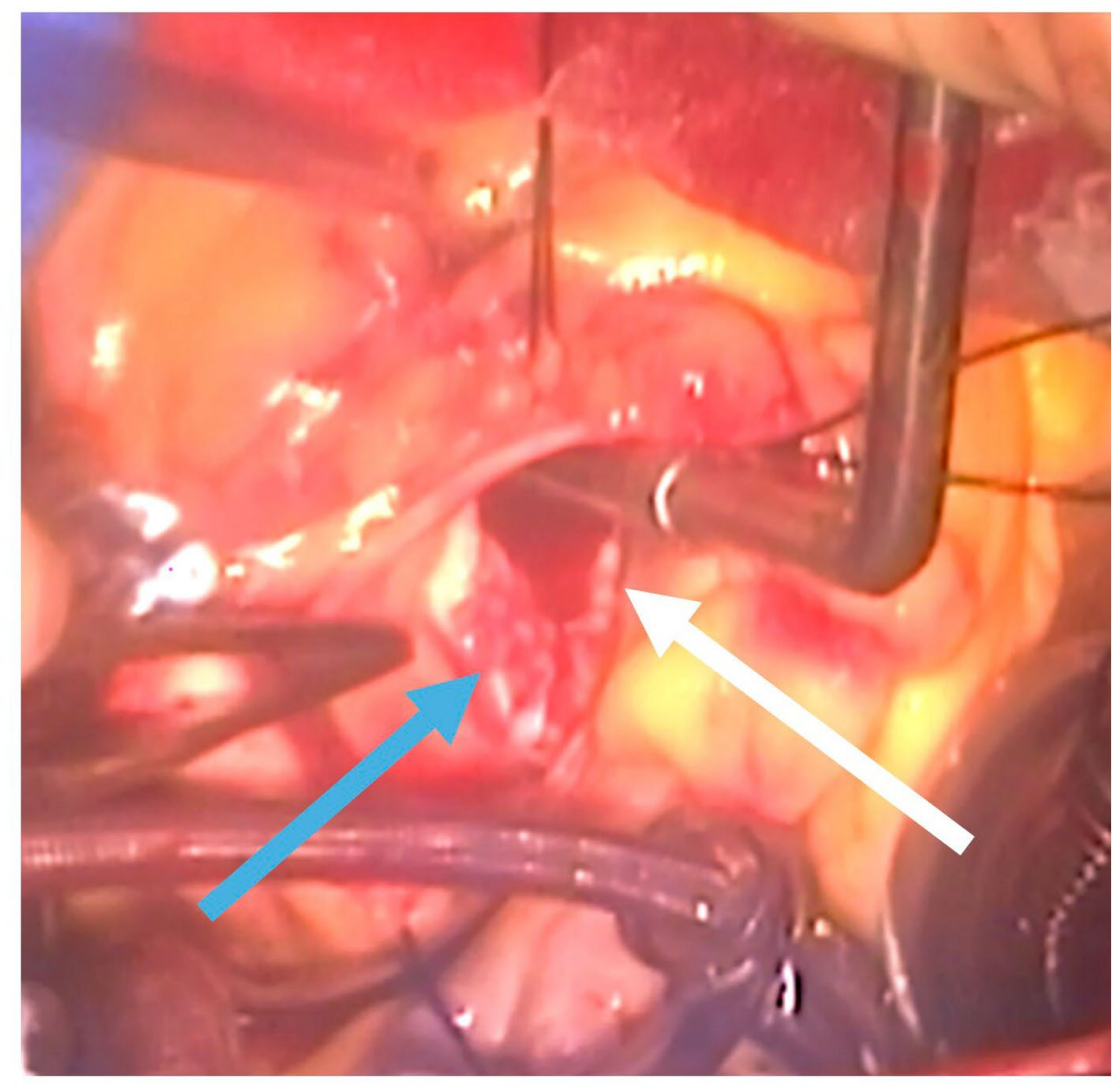
Intraoperative photograph of the explanted, degenerated 23 mm Sapien 3 prosthesis demonstrating leaflet thickening (blue arrow) and severe annular calcification (white arrow).

A median sternotomy approach was utilized with standard cardiopulmonary bypass cannulation. Following autotomy, the degenerated 23 mm Sapien 3 TAVR valve was identified with thickened and stenotic leaflets (Figure 2). The valve frame was excised using the rolling technique after a longitudinal cut.

Following TAVR explantation, the native aortic valve leaflets were excised. Assessment of the annulus revealed severe calcifications along the aortomitral curtain, which was the source of the paravalvular leak. A 5 mm × 25 mm felt patch was sutured along the aortomitral curtain under direct visualization.

**Figure 3:**
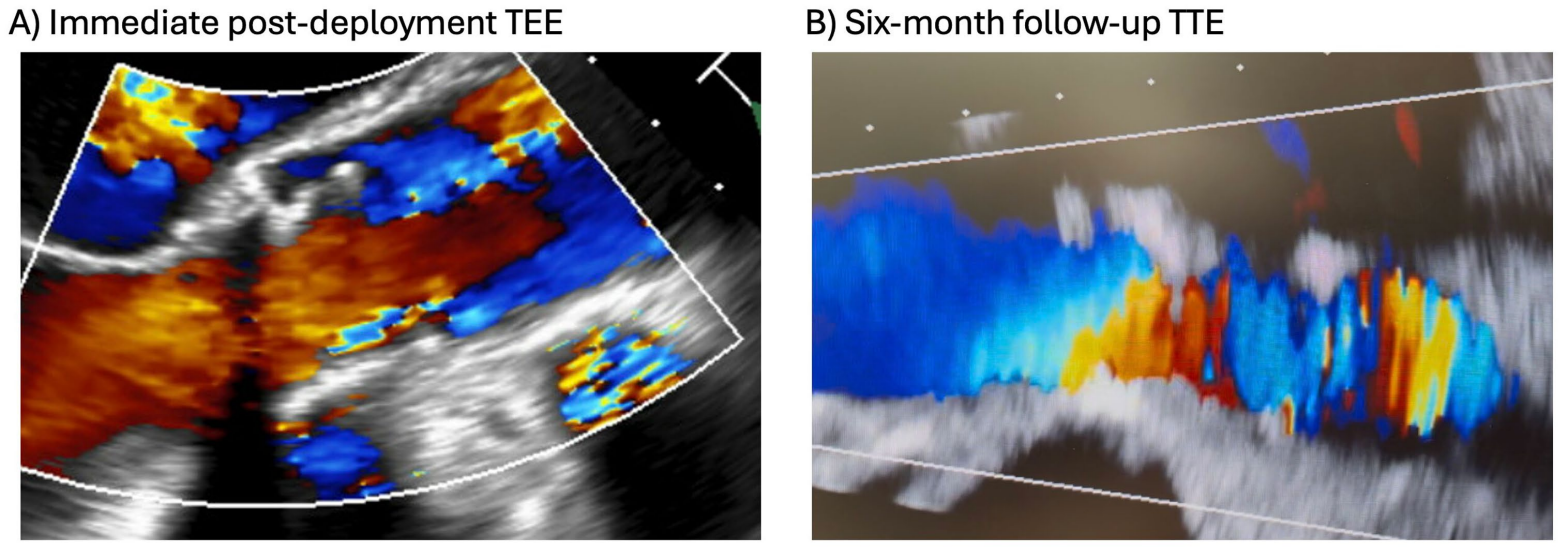
Two-panel figure showing **(A)** immediate post-deployment intraoperative TEE confirming preserved valve function and elimination of paravalvular leak, and **(B)** six-month follow-up TTE demonstrating sustained well-functioning bioprosthesis.

A 26 mm Sapien Ultra transcatheter valve was selected to optimize hemodynamics and address PPM. The new TAVR valve was deployed under direct visualization, ensuring precise coronary-valve alignment relative to the coronary ostia. *Novel technique:* After deployment of the 26 mm Sapien Ultra valve, the root was examined and noted to be overcrowded with the larger valve. The metal cells facing the left main coronary artery were then selectively excised using standard wire cutters to preserve future coronary access and facilitate any future percutaneous coronary intervention. Direct visualization confirmed adequate coronary access with an unobstructed view of the left main coronary ostium. Assessment with a saline test verified that valve function was not compromised (Figure 3).

The tricuspid valve was repaired with an MC3 annuloplasty band to address the moderate–severe tricuspid regurgitation. After cross-clamp removal, the heart resumed beating in sinus rhythm, and the patient demonstrated hemodynamic improvement. Post-surgery intraoperative transesophageal echocardiography verified resolution of stenosis, elimination of paravalvular leak, and adequate valve function.

The patient was transferred to the intensive care unit with standard postoperative monitoring. She was extubated on postoperative day 0 and progressed well. The hospital course was notable for paroxysmal atrial fibrillation with occasional bradycardia necessitating permanent pacemaker implantation on postoperative day 7. She was discharged home on postoperative day 8 in stable condition. The patient achieved immediate resolution of severe stenosis and elimination of PVL with hemodynamic improvement.

**Figure 4:**
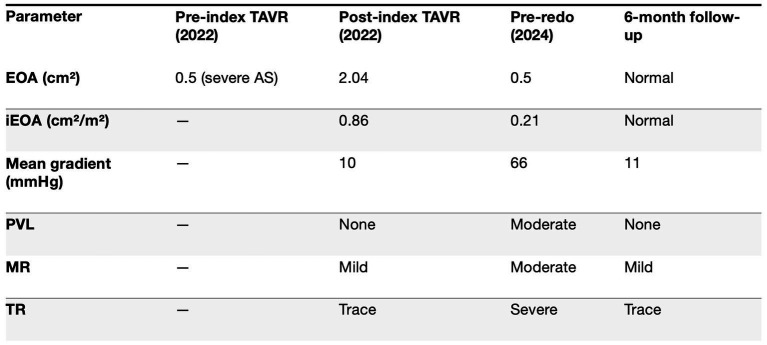
Hemodynamic trajectory across the patient’s TAVR course. The initial 23 mm Sapien 3 functioned well at implantation without patient–prosthesis mismatch (iEOA 0.86 cm^2^/m^2^) but underwent rapid structural valve degeneration over 2 years. The 26 mm Sapien Ultra implanted at the redo procedure demonstrated sustained function at six-month follow-up. EOA, effective orifice area; iEOA, indexed effective orifice area; MR, mitral regurgitation; PVL, paravalvular leak; TR, tricuspid regurgitation.

Six-month follow-up transthoracic echocardiography demonstrated a well-functioning bioprosthetic aortic valve with trace tricuspid regurgitation and improved mitral regurgitation (Figure 4). The prosthetic valve exhibited a mean transvalvular gradient of 11 mmHg, representing only a minimal rise from the immediate postoperative measurement. Should the newly implanted valve degenerate in the future, management options include valve-in-valve TAVR, now facilitated by the larger 26 mm platform, or repeat surgical explantation. Additionally, the prophylactic metal cell excision preserves the option for percutaneous coronary intervention should future coronary disease develop.

## 5 Discussion

Transcatheter aortic valve replacement has emerged as the dominant treatment for aortic stenosis, yet coronary access and long-term valve durability remain concerns. This case presents selective removal of TAVR metal cells facing the left main coronary artery during open valve implantation, directly addressing the challenge of post-TAVR coronary access.

Our approach offers several advantages over standard re-intervention options. The technique simultaneously corrects prosthesis–patient mismatch by upsizing from a 23 mm to a 26 mm valve, definitively repairs PVL with a targeted felt patch, and preserves future coronary access. Unlike traditional surgical aortic valve replacement, which carries 13–20% mortality, this hybrid approach minimized cardiac ischemia [88-min cross-clamp time for two valve procedures (9–11)]. Valve-in-valve TAVR, while less invasive, would have perpetuated prosthesis–patient mismatch (the constrained 19 × 19 mm internal diameter of the indwelling stent would have precluded meaningful hemodynamic improvement), and would not have definitively addressed the paravalvular leak or preserved coronary access.

It is important to distinguish two separate coronary concerns in this context: acute coronary obstruction at deployment and future selective catheter-based coronary engagement for percutaneous intervention. For this patient, the baseline risk for both was relatively low. Favorable annulus-to-coronary heights (11.1 mm left; 10.6 mm right) and sinus of Valsalva width (33.2 mm) were adequate to avoid acute obstruction during deployment, surgical excision of the native leaflets eliminated the dominant mechanism of acute coronary obstruction after TAVR, and the open-cell architecture of the Sapien Ultra frame typically permits coronary cannulation even when a strut overlies the ostium. Thus, the decision to proceed with metal cell excision to prophylactically optimize the predictability and ease of future catheter-based coronary engagement warrants careful examinations, particularly because frame modification raises legitimate concerns regarding radial force distribution, long-term durability, and frame integrity that intraoperative testing cannot assess.

When deciding to proceed with selective metal cell excision, four important points were considered. First, anatomic adequacy at the time of TAVR deployment does not equate to procedure ease for coronary cannulation. Engagement through a TAVR frame may require specialized techniques, which become more difficult in the setting of time-sensitive treatment of acute coronary syndrome. This patient’s coronary atherosclerotic disease (75% main coronary with multivessel involvement) supported the non-trivial likelihood of future coronary intervention. Second, deployment of the upsized 26 mm valve following explantation and native leaflet excision brought the frame into closer apposition with the aortic wall, raising the risk of sinus sequestration; selective excision of the metal cells facing the LMCA mitigated this immediate hemodynamic concern by creating a communication between the neo-sinus and aortic root, in addition to preserving future catheter-based access. Thirdly, in a known high-risk patient, the surgical setting provided a unique opportunity for well visualized frame modification that could not be safely performed once the chest was closed. Lastly, the conditions that allowed for modification (favorable anatomy, native leafelet removal, commissure alignment) allowed for a lower risk prophylactic procedure.

The rapid progression from a well-functioning prosthesis at implantation to severe stenosis and moderate PVL within 2 years warrants consideration of both patient-specific and population-level contributors. In this patient, preprocedural CT demonstrated a severe focal calcific arc (~90°) extending from the commissure between the left and noncoronary leaflets into the LVOT as a calcific ridge, a well-established anatomic risk factor for PVL, post-TAVR stroke, and increased hospital mortality (12–14). Additional patient-specific factors likely contributed to early SVD, including elevated BMI (46.2 kg/m^2^) and a relatively large BSA (2.36 m^2^) for a 23 mm prosthesis, which increases leaflet cyclic stress. Chronic kidney disease may have also played a role, which is a recognized accelerator of bioprosthetic leaflet calcification (15, 16). In the broader TAVR population, early SVD (occurring within 5 years) has been reported in 4–13% of patients and is increasingly recognized as TAVR expands to younger and lower-risk cohorts (17, 18). Additional established risk factors include smaller valve size, extensive or asymmetric calcification, diabetes mellitus, self-expandable valve, and elliptical annular shape (16). Recognition of these mechanisms is increasingly important as indications for TAVR expands.

Several limitations must be acknowledged. This represents a single-case experience with unknown long-term valve durability. When deemed necessary due to left main compromise, the optimal extent of safe metal cell removal remains undefined, and future studies must establish clear safety parameters. Hypothetical risks include radial force disruption that could affect valve durability, annular sealing, and leaflet stress. Exposed metal surfaces could hypothetically act as thrombogenic surfaces. Conduction disturbance requiring permanent pacemaker implantation is recognized after TAVR explantation, with reported rates of approximately 10–50%, owing to repeated mechanical injury to the membranous septum and AV node (19). In this patient, telemetry demonstrated paroxysmal atrial fibrillation with slow ventricular response and a junctional escape rhythm, with occasional wide-complex QRS beats following prolonged RR intervals, prompting pacemaker implantation on postoperative day 7. Metal cell excision was performed on the aortic side of the frame, distant from the LVOT-side cells overlying the conducting system. Given the known risk, it unlikely to have contributed directly; however, a theoretical relationship cannot be excluded. Lastly, efficacy with other transcatheter designs, particularly self-expanding platforms, remains uncertain.

To our knowledge, this is the first published description of selective metal cell excision during open TAVR implantation. While selective cell modification has been explored percutaneously post-TAVR (20), our approach differs fundamentally by performing modification at implantation with immediate verification. As TAVR expands to younger populations, techniques that preserve coronary access without compromising valve function become increasingly important, as these patients may require coronary interventions, valve re-intervention, or other cardiac procedures over the 20–30-year lifespan.

This case demonstrates that selective TAVR metal cell excision during open implantation is a technically feasible approach to preserve coronary access without compromising valve integrity or function. This technique also simultaneously addressed structural valve degeneration, prosthesis–patient mismatch, paravalvular leak, and multivalvular disease. By combining open surgical visualization with transcatheter valve technology, the approach captures hemodynamic benefits of surgical therapy while maintaining lower perioperative mortality. This technique warrants further investigation to validate durability, establish optimal safety parameters, and assess applicability across other valve platforms.

## 6 Patient perspective

Written informed consent was obtained from the patient for publication of this case report, including clinical details, diagnostic images, operative findings, and follow-up outcomes.

The patient reported significant improvement in her quality of life following the procedure. Prior to surgery, she experienced progressive shortness of breath that limited her activities of daily living. She understood that the selective metal cell excision technique was designed to preserve her options for future coronary procedures, and she expressed confidence in the surgical team’s decision-making process. At six-month follow-up, she reported sustained symptom improvement and satisfaction with the surgical outcome.

## Data Availability

The original contributions presented in the study are included in the article/Supplementary material, further inquiries can be directed to the corresponding author.
